# Assessment of pre-hospital emergency medical services in low-income settings using a health systems approach

**DOI:** 10.1186/s12245-018-0207-6

**Published:** 2018-11-22

**Authors:** Amber Mehmood, Armaan Ahmed Rowther, Olive Kobusingye, Adnan A. Hyder

**Affiliations:** 1 Johns Hopkins International Injury Research Unit, Health Systems Program, Department of International Health, Johns Hopkins Bloomberg School of Public Health, 615 N Wolfe St, Baltimore, MD 21205 USA; 2 Makerere University School of Public Health, Kampala, Uganda

**Keywords:** Emergency medical services, Pre-hospital care, Health services, Health system framework, Assessment, Instruments

## Abstract

Emergency medical services (EMS) is defined as the system that organizes all aspects of care provided to patients in the pre-hospital or out-of-hospital environment. Hence, EMS is a critical component of the health systems and is necessary to improve outcomes of injuries and other time-sensitive illnesses. Still there exists a substantial need for evidence to improve our understanding of the capacity of such systems as well as their strengths, weaknesses, and priority areas for improvement in low-resource environments. The aim was to develop a tool for assessment of the pre-hospital EMS system using the World Health Organization (WHO) health system framework. Relevant literature search and expert consultation helped identify variables describing system capacity, outputs, and goals of pre-hospital EMS. Those were organized according to the health systems framework, and a multipronged approach is proposed for data collection including use of qualitative and quantitative methods with triangulation of information from important stakeholders, direct observation, and policy document review. The resultant information is expected to provide a holistic picture of the pre-hospital emergency medical services and develop key recommendations for PEMS systems strengthening.

## Introduction

Injuries and other time-sensitive illnesses such as cardiac arrest, stroke, sepsis, and obstetric emergencies are significant contributors to premature mortality and disability in low- and middle-income countries (LMICs) [1, 2]. In these countries, the majority of early deaths from such time-sensitive conditions are the result of inadequate pre-hospital care, unavailability of transport, or both [1]. Patients may need to be transported more than 20 km to reach a health care facility in low-income countries, with up to 80% of them walking or being carried by family members [3]. Emergency medical services (EMS), which may encompass local, regional, or international systems for delivery of pre-hospital care, play a critical role in improving the outcomes of both acute diseases and acute exacerbations of chronic illnesses [4–8]. The evidence shows that the lack of pre-hospital care negatively affects the outcomes of medical, obstetric, and pediatric emergencies; the availability of pre-hospital care causes a 25% reduction in trauma-related mortality alone, with a larger cumulative effect when safe transport is combined with prompt facility-based emergency care [9, 10].

**Fig. 1:**
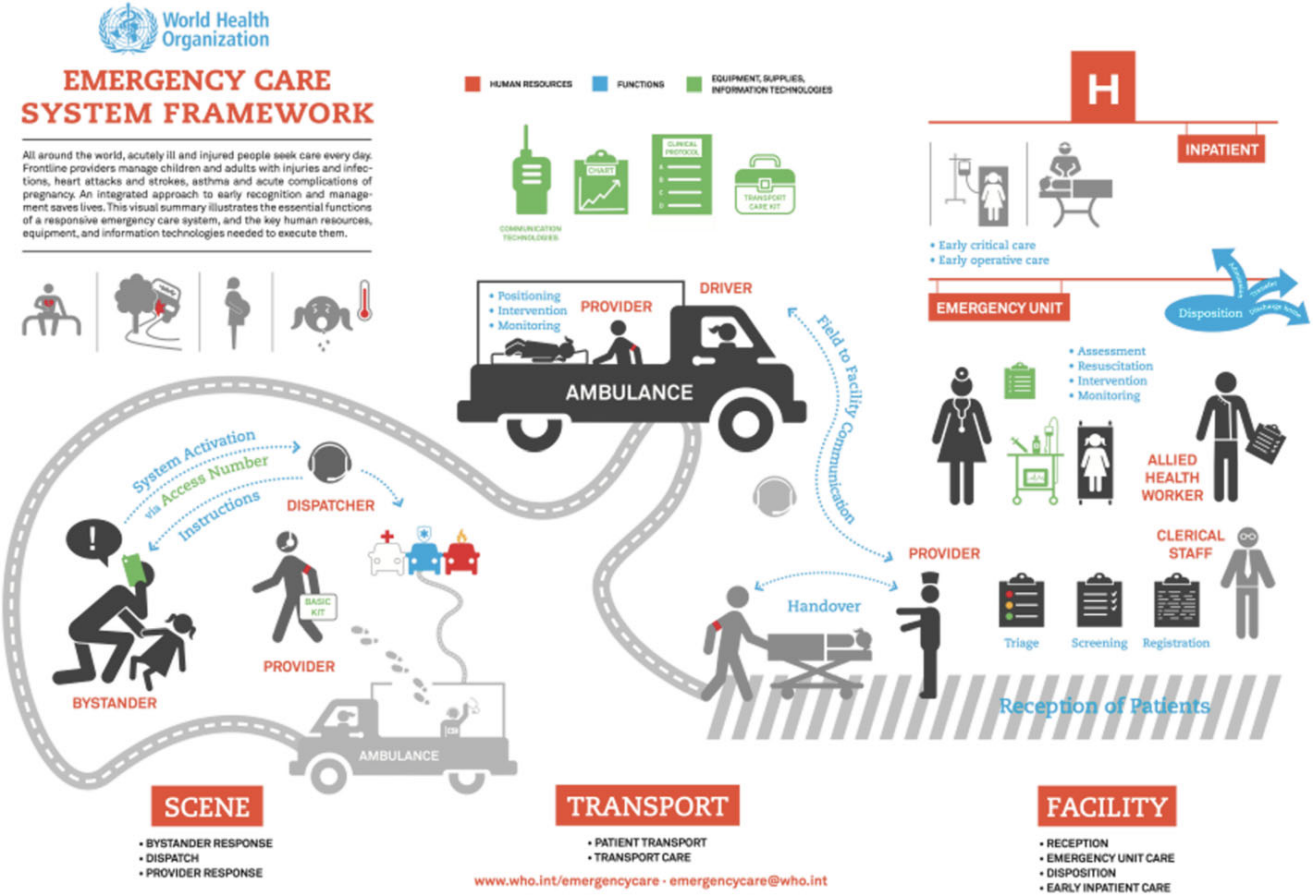
WHO emergency care system framework. This framework captures essential emergency care functions at the scene of injury or illness, during transport, and through to emergency unit and early inpatient care. Orange text and images represent human resources, blue represents system functions, and green represents equipment, supplies, and information technologies

EMS is defined as the system that organizes all aspects of medical care provided to patients in the pre-hospital or out-of-hospital environment (Fig. 1) [4, 11]. Generally speaking, patients requiring “pre-hospital care” are planned or intended to be transported to hospital for further treatment, whereas in “out-of-hospital” emergency care, such intent or planning may be absent [11]. Apart from being a common resource for a variety of medical conditions, EMS is also the foundation for effective disaster response and management of mass casualty incidents [12–14].

The organization and provision of EMS varies from country to country and sometimes between regions within a country. While many models of pre-hospital care are described in the literature from LMICs [15, 16], EMS systems are generally fragmented and largely limited to transportation without protocols for field triage, standards of care, or communication to receiving facilities. The lay public is often left to decide independently whether and where to transfer their acutely ill or injured patients.

In areas where EMS resources are limited to ambulance services for transporting patients with mild injuries and non-urgent illnesses, consumers sometimes perceive EMS as ineffective and inadequately equipped to deal with acute illnesses and life-threatening medical conditions [17–19]. Meanwhile, the true scope of EMS in LMICs, delivery of pre-hospital care, and the proportion of need being met remain known [20]. A recent landscape analysis demonstrated that less than one in three African countries has pre-hospital EMS in place, with limited capacity to respond, evaluate, treat, and safely transport patients [21].

Meeting a broad spectrum of medical needs requires innovative thinking, planning, and adaptation, particularly in areas with fewer resources. This issue was highlighted by the World Health Organization (WHO) in a 2007 resolution calling on national governments to strengthen emergency care globally in partnership with the WHO [22]. Despite a number of studies on EMS readiness, there is less evidence on the true capacity, performance, and sustainability of pre-hospital EMS (PEMS) systems. A thorough knowledge of infrastructure, service delivery, coverage, and information flow is required to determine if the PEMS system is sufficient to meet the health needs of a community [17, 18, 23].

There have been some efforts towards defining core elements, performance indicators, and gaps in service delivery for EMS and trauma care [20, 24, 25]. Some studies have addressed facility-based assessment based on WHO guidelines to identify gaps and prioritize areas for improvement in acute care facilities [18, 23, 26, 27]. However, there exists a need for a rapid yet comprehensive tool for systemic assessment of PEMS that combines input from the policymakers, care providers, and community members.

This paper describes the core elements of pre-hospital EMS (PEMS) system within the health systems framework and proposes a tool that focuses on system-wide assessment of PEMS in LMICs. The specific objectives of this paper include the following: (1) to provide a brief overview of selected instruments and approaches to PEMS assessment, (2) to identify PEMS-related variables and core indicators that provide information according to a health systems framework, and (3) to propose an approach for implementation of an assessment tool and identify sources of information for deployment in LMICs. This tool does not specifically address out-of-hospital and community-based emergency care, although the proposed framework covers broad components of the overall emergency medical care in LMICs [10].

## Select instruments and guidelines

There is a diverse body of published literature that covers PEMS, pre-hospital care standards, and international guidelines on the pre-hospital trauma care systems from high-income countries (HICs) and LMICs. The following section reviews key features of a select number of these guidelines and assessment instruments.

The American College of Surgeons (ACS) Committee on Trauma established guidelines for care of injured patients in the form of *resources for optimal trauma care*, which was first published in 1976 and is the foundation of the trauma center verification and certification process in the USA [28]. This policy document is a comprehensive resource inventory for high-quality facility-based trauma care that emphasizes the concept of an “inclusive” trauma system with well-defined assessment, verification, and performance improvement measures for trauma centers. These standards are difficult to achieve and maintain in many LMIC settings, however, and despite a public health model, this resource document narrowly focuses on trauma care as opposed to covering all emergencies.

The *WHO guidelines for essential trauma care*, developed in collaboration with the International Association for the Surgery of Trauma and Surgical Intensive Care in 2004, are directed towards improving facility-based trauma care and cover the knowledge, skills, and equipment required to deliver appropriate trauma care [29]. The guidelines include a series of resource tables for essential trauma care that detail the human and physical resources that should be in place at each health facilities, ranging from rural health posts, to hospitals staffed by general practitioners and specialists, to tertiary care centers. These guidelines also account for varying resource availability across the spectrum of LMICs. The document includes recommendations for training, performance improvement, and hospital inspection to optimize care of the injured but does not address other medical problems or common emergencies.

The *WHO guidelines for pre-hospital trauma care*, published in 2005, focus on standards of pre-hospital trauma care systems by providing a resource matrix with essential, desired, and possible components of knowledge, skills, equipment, and supplies, each classified according to the level of pre-hospital providers [30]. Since the key focus of these guidelines is to promote the development of pre-hospital trauma systems, its scope also extends to important system-level elements such as organization and oversight, coordination, documentation of care, and ethical and legal issues pertinent to trauma care. Generally, the *WHO guidelines for pre-hospital trauma care* stipulate the foundation for general emergency care and could be used for a broader range of emergencies, albeit with less specificity.

The most recently developed *WHO emergency care system assessment tool* has been designed to help policy-makers and planners assess a national or regional emergency care system, identify gaps, and set priorities for system development. It is a survey-based tool that can facilitate priority setting through convened external assessment [31]. This tool is also the most relevant to our study goals and objectives; however, the information input is largely dependent on the knowledge of key informants responding to survey questionnaires or in the setting of a convened consensus exercise. The survey does not include input from patients or customers of PEMS and hence allows gaps in assessment of access, quality, and responsiveness of services to remain.

A comparison of these instruments and guidelines from a health systems standpoint is summarized in Table 1.

**Table 1:** Description of available guidelines and instruments on pre-hospital and trauma care

	ACS resources for optimal trauma care	WHO guidelines for essential trauma care	WHO guidelines for pre-hospital trauma system	WHO emergency care system assessment tool
Stated purpose of the instrument	Guidelines for care of injured patients/trauma center verification	Guidelines to improve trauma care at facility level	Guidelines for planning and implementing trauma care in pre-hospital phase	To help policy-makers and planners assess a national or regional emergency care system
Unit	State/regional trauma system	Health care facilities of different level	Pre-hospital trauma system	Health system
Dimensions/input	Trauma center designation, pre-hospital care, transfer, clinical functions, trauma registry, performance improvement, disaster planning, etc.	14 aspects of clinical care including different injuries, diagnostic modalities, medicines, and rehabilitation, etc.	Seven key inputs, e.g., system models, providers, resource matrix, transport, communication, etc.	11 domains including trauma system organization, governance, financing, transport, facility-based care, surge capacity, etc.
Data collection methods	Onsite review of hospital for optimal resource verification	Survey or interview-based assessment	Survey or interview-based assessment	Survey of policy makers and administrators
Selected Indicators	Type I and type II indicators, where type I are deemed essential: patient safety, surgical functions, trauma patient volume, in-house attending	Knowledge/skill and equipment/ supplies for each category	Resource matrix including knowledge/skill and equipment/supplies	Lead agency, proportion of facilities with designated emergency unit, or 24-h dedicated staff, number of ambulances
Scope	Trauma center accreditation according to different levels of care	Assessment of level of care provided at each facility	Status of pre-hospital trauma care based on availability of essential, desirable and relevant skills and resources	Status of emergency care systems for policy and planning; identification of gaps and setting priorities for system development

## Conceptual framework for a PEMS assessment tool

To encompass a holistic picture of PEMS in a community or geographical locale, the “Framework for Action for strengthening health systems” proposed by the WHO provides essential domains of a system-based assessment [32]. The WHO health systems framework is designed to capture and quantify building blocks (inputs), outputs, and long-term outcomes. While long-term outcomes and impact of interventions may be difficult to measure in a cross-sectional assessment, the framework provides a guide for inclusion of important indicators.

**Fig. 2:**
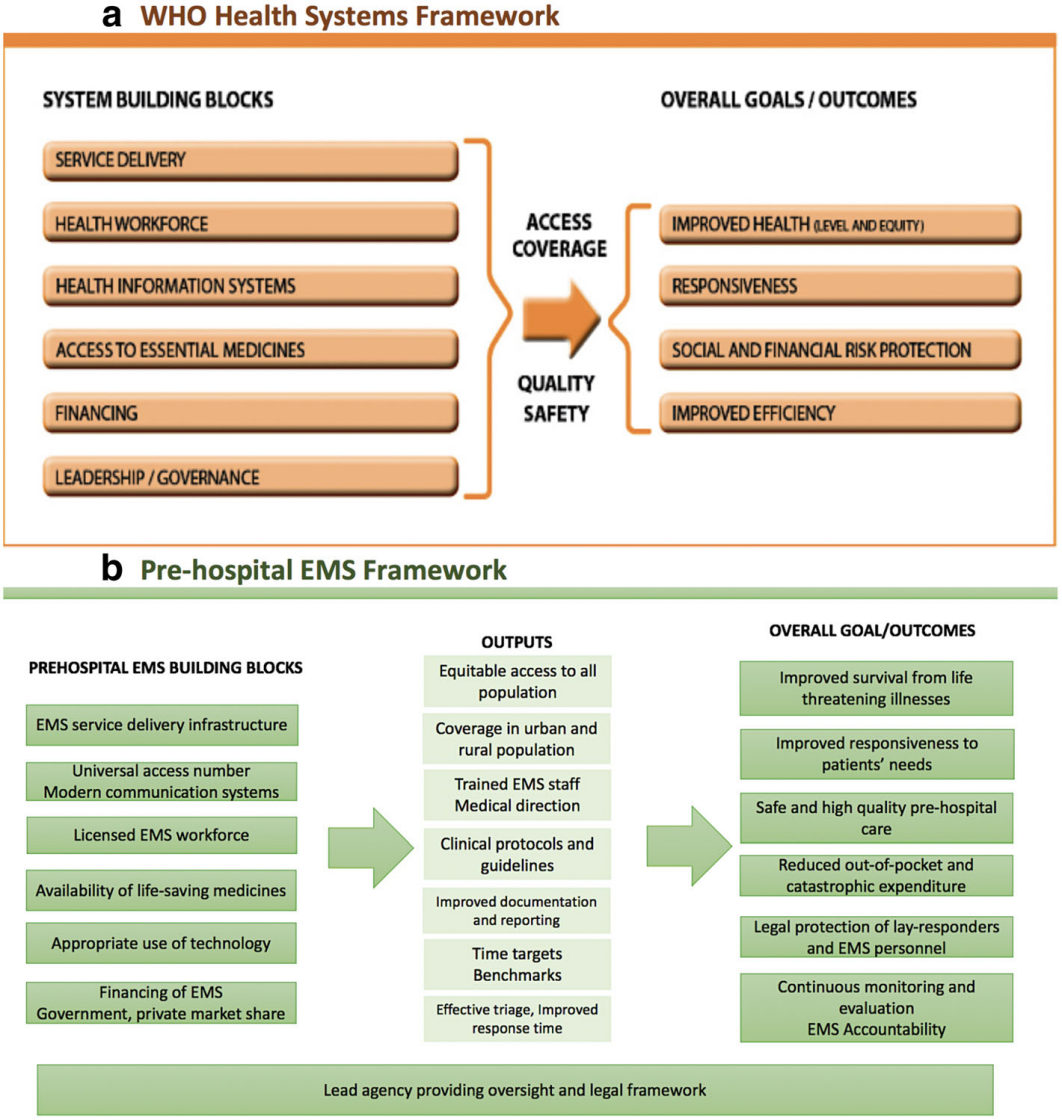
**a** WHO health systems framework. **b** Pre-hospital EMS framework. Using WHO health systems framework, pre-hospital care components are organized into building blocks of the EMS system to provide an evaluative framework for the assessment tool

This conceptual framework is supposed to rely upon the interrelatedness of (i) health service delivery model; (ii) well-performing, trained PEMS health workforce; (iii) well-functioning communication system that includes a Universal Access Number (UAN) and a dispatch system; (iv) access to life-saving medicines, equipment, procedures, and expertise at the scene, in transport, and during transfer; (v) appropriate use of technology by the PEMS staff and administrators; (vi) financing mechanisms to safeguard the sustainability of the PEMS, such as insurance coverage, incentives etc.; and (vii) leadership and governance that provide regulatory bodies as well as legal and policy frameworks. Building on the previous work, identification of variables and indicators of pre-hospital care could stipulate comprehensive information on inputs, processes, outputs, and desired outcome of PEMS [24, 33]. Figure 2 depicts the WHO health systems framework and key components that serve as building blocks, outputs, and goals of an EMS systems framework based on similar domains.

In a logic model, health systems inputs feed into processes and produce outputs that ultimately lead to an intended or desired outcome [34]. For instance, investment in PEMS services through universally accessible pre-hospital care, adequately trained personnel, and appropriate infrastructure would lead to a system that covers large populations and delivers high-quality care with safe clinical interventions to patients in a responsive and efficient manner using appropriate resources. The degree to which these services provide timely, skilled, and safe care through adherence to protocols, guidelines, and benchmarks could be measured through improved survival from acute life-threatening emergencies, reduction in out-of-pocket expenditure, and decreased mortality caused by delays in the pre-hospital phase. Overall outcomes may reflect the health systems’ ability to deliver PEMS services equitably and efficiently, which is critical for achieving improved health within the population it serves.

## Content of proposed assessment tool

**Fig. 3:**
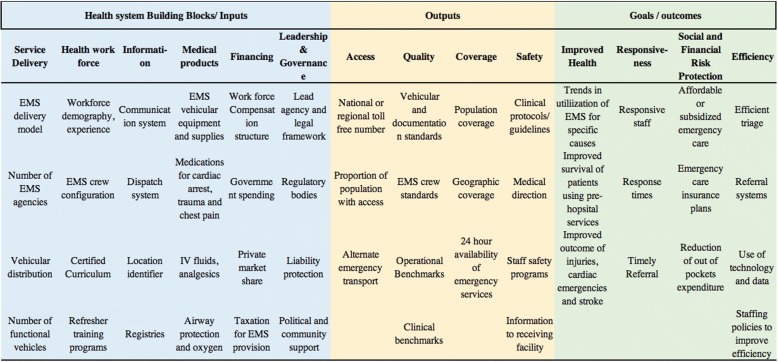
Domains of the prehospital framework. This figure outlines potential variables of the assessment tool to evaluate overall EMS system capacity and performance according to a health systems framework. Blue cells in the table represent system inputs, yellow represents system outputs, and green represents goals and outcomes

To ensure a sufficiently detailed and comprehensive system-wide assessment, we identified approximately 50 variables describing core components of system capacity. These include variables covering EMS building blocks, system outputs, and outcomes such as improved survival from life-threatening emergencies (Fig. 3). The variables were reviewed and finalized by a group of experts consisting of emergency physician, trauma surgeon, public health professionals, health system researchers, and EMS administrator. These experts were invited for review based on their familiarity with LMIC health systems, experience of providing emergency and trauma care in diverse settings, in-depth knowledge of the PEMS service delivery, and expertise in performance measurement and policy making. The variables were included based on the comprehensiveness of their scope, their ability to detect changes over time, and advances in EMS strengthening in LMICs (Fig. 3).

The proposed tool provides an adaptable template for national, regional, or city-wide EMS assessment. Input indicators include presence or absence of lead agencies, availability of Universal Access Number (UAN) and pre-hospital provider certification and licensing programs, number of registered EMS organizations, number of functional, fully-equipped vehicles, and other inputs that reflect capacity of the existing system to meet demand. Indicators for expenditures and methods of financing for emergency care including public funds, private insurance, and public-private partnerships help determine unmet needs or potential for financial risks for public, as well as planning for work-force compensation structure. The quality and safety of services would be measured through the availability of, and adherence to, protocols and guidelines for assessment, treatment, and transfer of patients from scene to health care facilities.

While many commonly used indicators in EMS are clinical or related to care processes, the scope of this assessment tool requires inclusion of appropriate system-based indicators, some of which are outlined in Table 2 [24]. The tool is flexible and depending upon the complexity of the system, baseline, or follow-up assessment of EMS, or evaluation of certain interventions, details, and indicators under each variable could be expanded. Although assessment of many such indicators may be limited in low resource settings by gaps in both quality and availability of data, their inclusion could help set up targets or feasibility studies and serve as the benchmarks for improvement toward systematic tracking of EMS strengthening progress.

**Table 2:** Domain-wise sample questions and indicators of EMS framework

No.	Domain	Sample questions/indicator
1	EMS service delivery	• Is the service organized at state level or national level? • Number of organizations registered for EMS delivery • Distribution of vehicles • Basic vs. advance life support services
2	Health workforce	• Is there certified training curriculum and licensing mechanism? • Average experience and turn over in EMS organizations • EMS crew configuration • EMS workforce demography (sex and age composition)
3	Information systems	• Radio communication, dispatch system • GPS trackers, location identifiers • Transfer of information from ambulance to receiving service
4	Medical products	• Availability of life-saving medications • Availability of aspirin, analgesia, oxygen in the vehicle
5	Financing	• Proportion of EMS covered by public funds • Do private insurance companies cover emergency care and EMS? • Other tax mechanisms to provide for EMS funds
6	Governance	• Is there a lead agency? • What is the legislative framework? • Are their regulatory bodies for setting and monitoring EMS standards?
7	Access	• Availability of Universal access number • Availability of alternate emergency services
8	Coverage	• Proportion of population covered by the ambulance services • Are there differences in coverage in rural vs. urban areas? • Proportion of towns or villages covered through EMS services all the time
9	Quality	• Use of standardized documentation • Audits, benchmarks, and indicators to track performance and outcomes, reporting frequency • Quality improvement initiatives • Standards for ambulance and crew configuration
10	Safety	• Availability of clear guidelines, protocols for assessment, treatment, and transfer; hands-off protocols • Medical direction • Working conditions; injury prevention policies and working hours of the ambulance crew • Enforcement of regulations
11	Improved Health	• Trends in pre-hospital survival of select emergency condition for ambulance- transported patients
12	Responsiveness	• Responsiveness of EMS services, average response time
13	Risk protection	• Availability of emergency care insurance plans through employers or private companies • Out-of-pocket expenditure for EMS care
14	Improved efficiency	• Efficient field triage • Use of technology and data to improve services such as real-time electronic run sheets

## Administration of the tool—proposed approach

Health systems research frequently employs mixed methods, contextual knowledge, and triangulation of information to ensure methodological rigor, even to answer programmatic and operational questions [35]. Hence, a multi-pronged approach is recommended to cover a wide range of information and explore contextual details about services delivery, access, and quality of care. A team experienced in conducting surveys and in-depth interviews coupled with local knowledge and access to communities would be able to conduct this type of assessment swiftly. Proposed data collection activities and data sources are summarized in Table 3 and are described in detail below.

**Table 3:** Domains of PEMS framework and proposed data collection strategy

Tool	Document review	Inspection/observation	Key informant interviews	Focus group discussion
Service delivery	√		√	
Health workforce	√	√	√	
Information	√	√	√	
Medical products	√	√	√	
Financing	√		√	
Governance/leadership	√		√	√
Access			√	√
Coverage	√		√	
Quality	√	√	√	√
Safety	√	√	√	√
Improved health	√		√	√
Responsiveness		√		√
Financial and social risk protection			√	√
Efficiency	√	√	√	

### Review of secondary data sources

Depending on duration of formal EMS services availability of relevant data from existing information systems, a number of secondary data sources could be examined. This may include document reviews to understand the legislative framework, vehicular standards, clinical protocols, medical direction, and training requirements, curriculum standards, and credentialing procedures. Information from secondary data sources could be used provide a snapshot of the PEMS system through workforce demography; geographic coverage; breakdown of PEMS response by time, location, and primary medical complaints; and insights into the evolution of services through average response times, trends in coverage, and staff turnover. Some sources that could provide such information are described below:

- National and regional policy documents for relevant EMS legislation, including Good Samaritan Laws, to provide basic legal protection for those who assist an injured person or provide care in medical emergencies; guidelines for lead organization, governance structures, agreements for coordination between agencies and financing mechanisms for PEMS and hospital-based emergency care. An effort should be made to obtain documents spanning 4 to 5 years to track policy changes or recent developments.
- Ambulance and PEMS provider organization documents and public reports could be obtained to review coverage of pre-hospital emergency services, workforce distribution, documentation standards, triage protocols, clinical guidelines, hands-off procedure, referral methods, and protocols.
- Curriculum, training, and licensing requirements for emergency medical technicians or paramedics working in pre-hospital environment. The source of this information could be individual PEMS provider organizations or a formal training institution certified by an education board or equivalent.
- Annual or quarterly reports of PEMS organizations, audit reports, EMS call logs, or registers to review nature and volume of emergency calls. An effort should be made to obtain reports spanning 3 to 5 years, if available, to allow for a trends analysis.

### Survey of ambulances and PEMS stations

Direct non-participant observation of representative sample of ambulance vehicles, ambulance stations, and dispatch centers covering major PEMS organizations would be conducted to triangulate information obtained through document review with direct observation of infrastructure, and objectively assess vehicular standards, available equipment and medications, communication systems, and documentation practices. PEMS system capacity to handle common emergency conditions including acute chest pain, traumatic injury, obstetric emergencies, and respiratory distress would be assessed using infrastructure checklists. Checklist components would cover equipment, supplies, protocols, and personnel basic knowledge of these conditions. Working conditions, safety of the staff, and dispatch systems could be directly observed at the ambulance stations and dispatch centers.

### In-depth interviews

This aspect of system assessment would seek to describe the PEMS system with specific attention to identifying formal and informal systems, insights about implementation of the policies and regulations, and perceptions of the community and pre-hospital staff. Qualitative data collection methods to be used would include:

- Key informant interviews (KIIs) to elicit information regarding policy and implementation gaps, financial sustainability, mass casualty management capabilities, internal and inter-agency coordination, communication procedures, work force safety procedures, and knowledge and practices of the care providers. Generally, the respondents could be selected using maximum variation purposive sampling to allow the capture of information pertaining to one or more building block, as well as management, operations, and associated support functions. These include but not limited to, policy makers, health system planners, PEMS administrators, and ambulance staff, identified with the help of governmental or pre-hospital care organizations [36]. KIIs would be conducted through semi-structured questionnaires with response guides for data collectors including follow-up questions. The sample size would vary depending upon the scale and aims of assessment, as described previously.
- Focus group discussions (FDGs) with community members is conducted to understand perceptions, opinions, beliefs, and attitudes towards access to pre-hospital, barriers to PEMS utilization, responsiveness of services, and quality of care during transport. FGDs could also explore the magnitude of financial burden and mechanisms to cover out-of-pocket expenditure among different customers. FGDs with first responders (e.g., police), emergency room physicians, or staff of major hospitals, who receive patients transported by ambulance services, could provide information about their challenges and perception about standard of care and safety PEMS during patient transport.

Quantitative results from direct observation and secondary data sources would be used to determine the proportion of ambulance services, organizations, or supplies meeting the minimum standards for each indicator, or categorical responses. Examples include “80% EMS organizations conduct annual audits,” “5/8 ambulances have functional airway equipment,” or “no official ambulance standards” where applicable.

It is recommended that KIIs and FGDs must be audio recorded if possible; notes and summaries documenting important findings, observations, and issues should be maintained. Regular debriefing meetings after qualitative interviews and discussions help in review of information and planning for further data collection. Comparing transcripts with audio files and summaries helps in monitoring quality of data. For qualitative data, a thematic analysis would be conducted using commercially available ATLAS-ti® or NVivo® software. A code book with a priori codes could be useful in understanding issues related to access to pre-hospital care, public’s attitudes, and perceptions about EMS in their respective environment and quality of services. Open and axial coding helps to conceptualize and label data from KIIS or FGDs that would be subsequently grouped into categories and sub-themes [37]. Simultaneous data collection and summarization of information helps in identifying the point where saturation is achieved. Based on this approach, the tool was initially validated in Uganda, where a limited PEMS assessment of the Kampala city was conducted, and the results were used to refine the questionnaire and identify solutions to the common logistical barriers during implementation [38].

## Advantage and limitations of the proposed tool

The ability to identify contextual barriers in pre-hospital care services in different countries, local definitions of “emergency,” and barriers to seeking care may vary in different communities [19]. Gaps and weaknesses in the infrastructure, resources, or health workforce have been studied previously in LMIC settings, but from an input perspective [16].

While the WHO health system framework is valuable because of its simplicity, segmented analysis of resources as building blocks without accounting for interaction among inputs may impact understanding of the process and outcomes [39]. Our proposed approach could overcome this limitation by incorporating quantitative and qualitative measures, triangulating data, and including secondary data spanning over a period of time. Participation of EMS providers and community members will ensure that the system’s resources, processes, and outputs are adequately compared against the perceptions and expectations of these stakeholders [3]. In conducting a comprehensive assessment, logistical delays must be kept in mind and could be overcome by pre-implementation planning. Early engagement with the stakeholders would expedite the approval processes needed for obtaining secondary data and organization of KIIs and FGDs. It is important to note that the tool is designed for a comprehensive system-wide PEMS assessment, not for service delivery monitoring purposes. In case a follow-up assessment is planned, it is recommended that such assessment would be undertaken with a gap of at least 3–4 years, to give sufficient time for implementing new system-based interventions, followed by evaluation of the expected outcomes. The tool covers the entire framework of PEMS; therefore, it is possible to deploy the tool for specific parts which are the focus of planning or evaluation of an intervention instead of conducting a comprehensive assessment. For instance, FGDs highlight the access, financial barriers, responsiveness, and quality of PEMS; document review to observe trends of service delivery and outcome indicators, etc. We have demonstrated that such an assessment could be done by local team including public health practitioners, without extensive guidance from international experts. Most of the document reviews and KIIs are conducted through semi-structured questionnaire, and observational study through checklists. FGDs are the only component that requires training and qualitative data analysis skills. Given the fact that neither this method is uncommon in LMICs nor underutilized in answering programmatic and operational questions, it is expected that local teams would be able to conduct the assessment successfully.

## Conclusion

This paper describes a comprehensive EMS assessment tool with a solid health system foundation. This tool could enable researchers, policy makers, and administrators alike to apply rigorous methods of PEMS assessment and use the information to set up and monitor benchmarks of health outcomes impacted by an organized EMS system. This tool provides a comprehensive health systems framework and at the same time carries the flexibility to focus on different parts of the PEMS where LMICs could focus, set their own benchmarks, and monitor their progress.

## Abbreviations

ACS: American College of Surgeons
EMS: Emergency medical services
EMT: Emergency medical technician
FGD: Focus group discussion
HIC: High-income country
KII: Key informants’ interview
LMIC: Low- and middle-income country
WHO: World Health Organization
